# Supplementary material for: A massively parallel CRISPR-based screening platform for modifiers of neuronal depolarization
Source: Nat Commun. 2026 Aug 1;17:9299. doi: 10.1038/s41467-026-75882-0 (PMC13526836; doi:10.1038/s41467-026-75882-0)
Supplement: Supplementary file 1 [file 41467_2026_75882_MOESM11_ESM.pdf]

nature portfolio

Corresponding author(s): Martin Kampmann
Last updated by author(s): Jul 11, 2026

Reporting Summary

Nature Portfolio wishes to improve the reproducibility of the work that we publish. This form provides structure for consistency and transparency in reporting. For further information on Nature Portfolio policies, see our Editorial Policies and the Editorial Policy Checklist.

Statistics

For all statistical analyses, confirm that the following items are present in the figure legend, table legend, main text, or Methods section.

n/a	Confirmed	
☐	☒	The exact sample size (*n*) for each experimental group/condition, given as a discrete number and unit of measurement
☐	☒	A statement on whether measurements were taken from distinct samples or whether the same sample was measured repeatedly
☐	☒	The statistical test(s) used AND whether they are one- or two-sided<br>*Only common tests should be described solely by name; describe more complex techniques in the Methods section.*
☒	☐	A description of all covariates tested
☐	☒	A description of any assumptions or corrections, such as tests of normality and adjustment for multiple comparisons
☐	☒	A full description of the statistical parameters including central tendency (e.g. means) or other basic estimates (e.g. regression coefficient) AND variation (e.g. standard deviation) or associated estimates of uncertainty (e.g. confidence intervals)
☐	☒	For null hypothesis testing, the test statistic (e.g. *F*, *t*, *r*) with confidence intervals, effect sizes, degrees of freedom and *P* value noted<br>*Give P values as exact values whenever suitable.*
☒	☐	For Bayesian analysis, information on the choice of priors and Markov chain Monte Carlo settings
☒	☐	For hierarchical and complex designs, identification of the appropriate level for tests and full reporting of outcomes
☐	☒	Estimates of effect sizes (e.g. Cohen's *d*, Pearson's *r*), indicating how they were calculated

Our web collection on statistics for biologists contains articles on many of the points above.

Software and code

Policy information about availability of computer code

Data collection	For FACS data collection, we used BD FACSDiva (version 8.0.1.1). For microscopy data collection by Image Express Confocal HT.ai, we used MetaXpress Acquisition Software. For qPCR data collection, we used Quant Studio Real Time PCR software (version 1.3). For electrophysiology recordings, we used pClamp10 acquisition software (Molecular Devices).
Data analysis	For FACS analysis, we used FlowJo (version 10.10.0) and Prism 10 (10.1.1). For microscopy image analysis we used CellProfiler (version 4.2.4). For pooled CRISPR screen analysis, we used fxtools, sgcount, and crispr_screen.<br>The sgcount, and crispr_screen bioinformatics pipeline for analysis of pooled screens available at https://kampmannlab.ucsf.edu and on Github at https://github.com/noamteyssier. The CellProfiler pipelines will be made available on request to the corresponding authors (MK), and will also be submitted to the CellProfiler depository of published pipelines (https://cellprofiler.org/examples/published_pipelines.html) upon publication.

For manuscripts utilizing custom algorithms or software that are central to the research but not yet described in published literature, software must be made available to editors and reviewers. We strongly encourage code deposition in a community repository (e.g. GitHub). See the Nature Portfolio guidelines for submitting code & software for further information.

Data

Policy information about availability of data

All manuscripts must include a data availability statement. This statement should provide the following information, where applicable:

- Accession codes, unique identifiers, or web links for publicly available datasets
- A description of any restrictions on data availability
- For clinical datasets or third party data, please ensure that the statement adheres to our policy

All screen datasets will be shared on the CRISPRbrain data commons (http://crisprbrain.org/) and will be shared upon request (associated with Figs. 2 and 4 and Supplementary Figure. 2). Single-cell RNA sequencing data is available through NCBI GEO (GSE289235)..

The CaMPARI2 screen counts data generated in this study will deposited in the CRISPRbrain data commons (http://crisprbrain.org/) and will also be shared upon request (associated with Figs. 2 and 4 and Supplementary Figure. 2).

The CROP-seq data generated in this study have been deposited in the NCBI GEO () under accession code GSE289235 https://www.ncbi.nlm.nih.gov/geo/query/acc.cgi?acc=GSE289235.

There are no restrictions on data availability. Source data are provided with this paper.

Research involving human participants, their data, or biological material

Policy information about studies with human participants or human data. See also policy information about sex, gender (identity/presentation), and sexual orientation and race, ethnicity and racism.

Reporting on sex and gender	*Use the terms sex (biological attribute) and gender (shaped by social and cultural circumstances) carefully in order to avoid confusing both terms. Indicate if findings apply to only one sex or gender; describe whether sex and gender were considered in study design; whether sex and/or gender was determined based on self-reporting or assigned and methods used. Provide in the source data disaggregated sex and gender data, where this information has been collected, and if consent has been obtained for sharing of individual-level data; provide overall numbers in this Reporting Summary. Please state if this information has not been collected. Report sex- and gender-based analyses where performed, justify reasons for lack of sex- and gender-based analysis.*
Reporting on race, ethnicity, or other socially relevant groupings	*Please specify the socially constructed or socially relevant categorization variable(s) used in your manuscript and explain why they were used. Please note that such variables should not be used as proxies for other socially constructed/relevant variables (for example, race or ethnicity should not be used as a proxy for socioeconomic status). Provide clear definitions of the relevant terms used, how they were provided (by the participants/respondents, the researchers, or third parties), and the method(s) used to classify people into the different categories (e.g. self-report, census or administrative data, social media data, etc.) Please provide details about how you controlled for confounding variables in your analyses.*
Population characteristics	*Describe the covariate-relevant population characteristics of the human research participants (e.g. age, genotypic information, past and current diagnosis and treatment categories). If you filled out the behavioural & social sciences study design questions and have nothing to add here, write "See above."*
Recruitment	*Describe how participants were recruited. Outline any potential self-selection bias or other biases that may be present and how these are likely to impact results.*
Ethics oversight	*Identify the organization(s) that approved the study protocol.*

Note that full information on the approval of the study protocol must also be provided in the manuscript.

Field-specific reporting

Please select the one below that is the best fit for your research. If you are not sure, read the appropriate sections before making your selection.

☒ Life sciences ☐ Behavioural & social sciences ☐ Ecological, evolutionary & environmental sciences

For a reference copy of the document with all sections, see nature.com/documents/nr-reporting-summary-flat.pdf

Life sciences study design

All studies must disclose on these points even when the disclosure is negative.

Sample size	Sample sizes were determined by existing studies in the field to enable statistical analyses and reproducibility, specifically our previous CRISPRi screens in iPSC-derived neurons (Tian et al. 2019, Neuron 104:239-255).
Data exclusions	No data was excluded from the relevant analyses.

Replication	Major findings were validated using independent samples and orthogonal approaches. Numbers of replicates are listed in each Figure.
Randomization	No randomization of samples was used since treatment group of cells were generally derived from the same population of cells.
Blinding	Blinding was not relevant to our study since no subjective rating of data was involved. Instead, we used computational quantification.

Reporting for specific materials, systems and methods

We require information from authors about some types of materials, experimental systems and methods used in many studies. Here, indicate whether each material, system or method listed is relevant to your study. If you are not sure if a list item applies to your research, read the appropriate section before selecting a response.

Materials & experimental systems

n/a	Involved in the study
☒	☐ Antibodies
☐	☒ Eukaryotic cell lines
☒	☐ Palaeontology and archaeology
☒	☐ Animals and other organisms
☒	☐ Clinical data
☒	☐ Dual use research of concern
☒	☐ Plants

Methods

n/a	Involved in the study
☒	☐ ChIP-seq
☐	☒ Flow cytometry
☒	☐ MRI-based neuroimaging

Eukaryotic cell lines

Policy information about cell lines and Sex and Gender in Research

Cell line source(s)	The human iPSC line WTc11 was obtained from Coriell (GM25256). The iPSC line used for electrophysiological characterization was obtained from Alstem (cat no iP11N). HEK293Ts were obtained from ATCC (CRL-3216).
Authentication	iPSCs were characterized for normal karyotypes and for maintaining capability to differentiate into glutamatergic neurons. We did not authenticate the HEK293T cells.
Mycoplasma contamination	All lines were tested negative for mycoplasma using the Universal Mycoplasma Detection Kit (ATCC 30-1012KTM).
Commonly misidentified lines (See ICLAC register)	No commonly misidentified lines were used.

Plants

Seed stocks	*Report on the source of all seed stocks or other plant material used. If applicable, state the seed stock centre and catalogue number. If plant specimens were collected from the field, describe the collection location, date and sampling procedures.*
Novel plant genotypes	*Describe the methods by which all novel plant genotypes were produced. This includes those generated by transgenic approaches, gene editing, chemical/radiation-based mutagenesis and hybridization. For transgenic lines, describe the transformation method, the number of independent lines analyzed and the generation upon which experiments were performed. For gene-edited lines, describe the editor used, the endogenous sequence targeted for editing, the targeting guide RNA sequence (if applicable) and how the editor was applied.*
Authentication	*Describe any authentication procedures for each seed stock used or novel genotype generated. Describe any experiments used to assess the effect of a mutation and, where applicable, how potential secondary effects (e.g. second site T-DNA insertions, mosiacism, off-target gene editing) were examined.*

Flow Cytometry

Plots

Confirm that:

☒ The axis labels state the marker and fluorochrome used (e.g. CD4-FITC).

☒ The axis scales are clearly visible. Include numbers along axes only for bottom left plot of group (a 'group' is an analysis of identical markers).

☒ All plots are contour plots with outliers or pseudocolor plots.

☒ A numerical value for number of cells or percentage (with statistics) is provided.

Methodology

Sample preparation	Cells were dissociated using Papain (for neurons) or Accutase (for iPSCs) and analyzed or sorted by flow cytometry based on staining signals. All stains were performed according to manufacturing protocols.Dissociation solution for neurons was made from resuspending papain (Worthington; Code: PAP2; Cat. No.LK003178) in 1X Hank's Balanced Salt Solution (Corning; Cat. No. 21-022-CV) to 20 U/mL. 5 mM $MgCl_2$ and 10 U/mL DNaseI (Worthington; Code: DPRF; Cat. No. LS006333). Dissociating solution was added to 96-well, 24-well, 12-well, 6-well plates and 15 cm dishes at volumes of 20 µL, 100 µL, 250 µL, 500 µL, and 5 mL, respectively. iNeurons were treated with dissociation solution for 12 minutes at 37ºC and resuspended in 1X Hank's Balanced Salt Solution (HBSS, Gibco) before analysis.<br>CaMPARI2 iNeurons plated on poly-D-lysine coated cell culture plates were illuminated with 405 nm light (LED output 39 W) in a temperature-controlled box with rotating turntable (FormLabs FH-CU-01).
Instrument	BD LSRFortessa X14 (BD Biosciences)
Software	Aquisition software used: BD FACSDiva (v.8.0.1.1). Flow cytometry data was analyzed using FlowJo analysis software (v10.10.0; BD Life Sciences
Cell population abundance	In the experiments used to analyze CaMPARI2 signal, cells were analyzed and not sorted. The starting populations were pure (either neurons or iPSCs) For pooled screens, these cells were sorted into high and low signal populations corresponding to the top 35% and the bottom 35% of the CaMPARI2 signal distribution.
Gating strategy	Preliminary FSC/SSC gates encompassed all live, single cells in the population. For pooled screens, these cells that were positive for sgRNA (BFP) and CaMPARI2 expression were sorted into high and low signal populations corresponding to the top 35% and the bottom 35% of the CaMPARI2 ratio distribution. For validation experiments, sgRNA+ and sgRNA- (based on BFP expression) populations were analyzed by the mean CaMPARI2 ratio signal.

☒ Tick this box to confirm that a figure exemplifying the gating strategy is provided in the Supplementary Information.
