# Supplementary material for: A massively parallel CRISPR-based screening platform for modifiers of neuronal depolarization
Source: Nat Commun. 2026 Aug 1;17:9299. doi: 10.1038/s41467-026-75882-0 (PMC13526836; doi:10.1038/s41467-026-75882-0)
Supplement: Supplementary file 2 [file 41467_2026_75882_MOESM1_ESM.pdf]

A Massively Parallel CRISPR-Based Screening Platform for Modifiers of Neuronal Depolarization

Boggess et al.

Supplementary Information

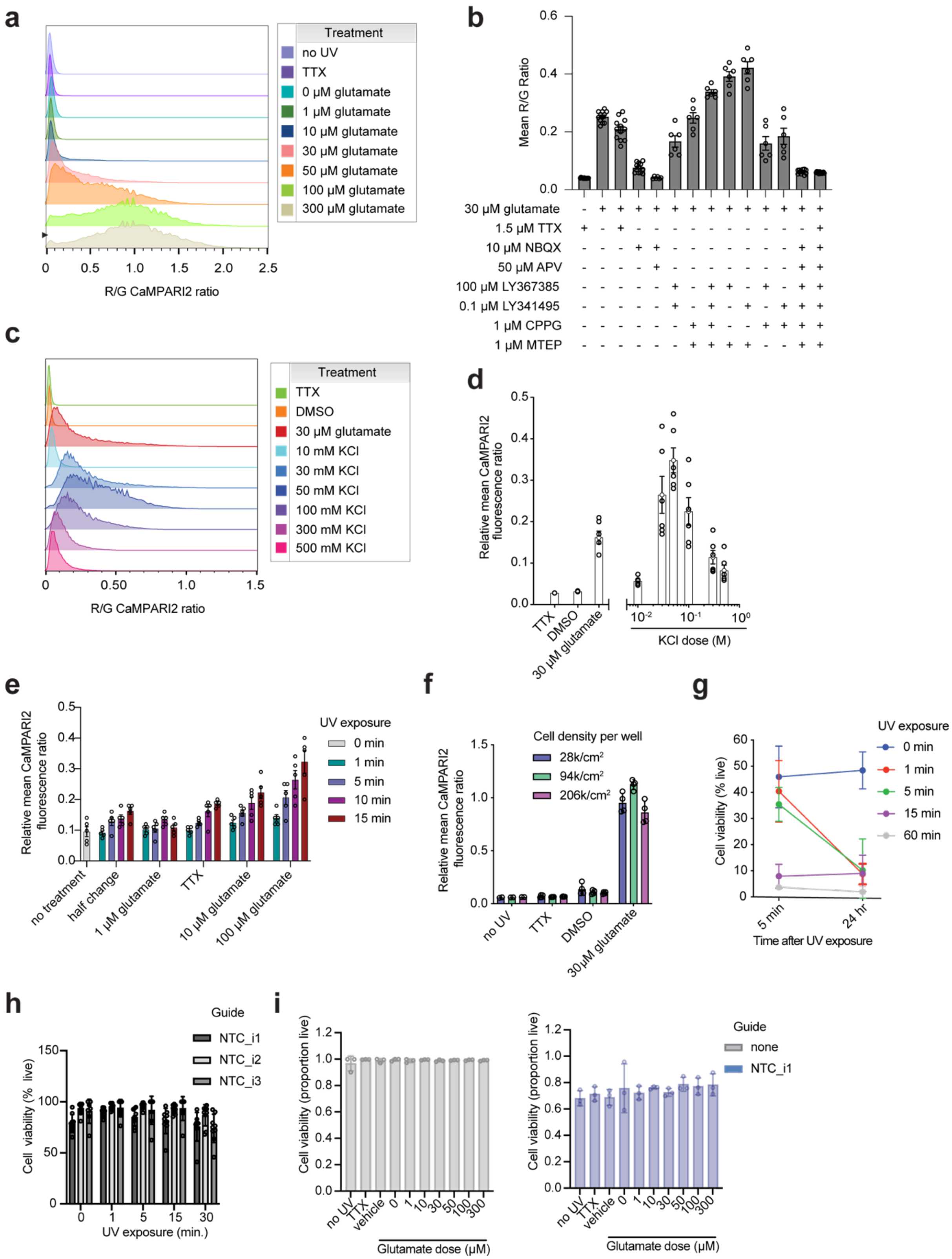
a
Treatment
no UV
TTX
0 μM glutamate
1 μM glutamate
10 μM glutamate
30 μM glutamate
50 μM glutamate
100 μM glutamate
300 μM glutamate
0 0.5 1.0 1.5 2.0 2.5
R/G CaMPARI2 ratio
b
Mean R/G Ratio
0.0 0.2 0.4 0.6
30 μM glutamate
1.5 μM TTX
10 μM NBQX
50 μM APV
100 μM LY367385
0.1 μM LY341495
1 μM CPPG
1 μM MTEP
c
Treatment
TTX
DMSO
30 μM glutamate
10 mM KCl
30 mM KCl
50 mM KCl
100 mM KCl
300 mM KCl
500 mM KCl
0 0.50 1.0 1.5
R/G CaMPARI2 ratio
d
Relative mean CaMPARI2 fluorescence ratio
0.0 0.1 0.2 0.3 0.4 0.5
TTX
DMSO
30 μM glutamate
10⁻² 10⁻¹ 10⁰
KCl dose (M)
e
Relative mean CaMPARI2 fluorescence ratio
0.0 0.1 0.2 0.3 0.4 0.5
UV exposure
0 min
1 min
5 min
10 min
15 min
no treatment
half change
1 μM glutamate
TTX
10 μM glutamate
100 μM glutamate
f
Relative mean CaMPARI2 fluorescence ratio
0.0 0.5 1.0 1.5
Cell density per well
28k/cm²
94k/cm²
206k/cm²
no UV
TTX
DMSO
30μM glutamate
g
Cell viability (% live)
0 10 20 30 40 50 60
UV exposure
0 min
1 min
5 min
15 min
60 min
5 min
24 hr
Time after UV exposure
h
Guide
NTC_i1
NTC_i2
NTC_i3
Cell viability (% live)
0 50 100 150
0 1 5 15 30
UV exposure (min.)
i
Cell viability (proportion live)
0.0 0.2 0.4 0.6 0.8 1.0 1.2
no UV
TTX
vehicle
0 1 10 30 50 100 300
Glutamate dose (μM)
Guide
none
NTC_i1

Supplementary Figure 1. CaMPARI2 reliably conveys AMPAR activation at the synapse without significant UV toxicity.

(a) CaMPARI2 dose response to glutamate. CaMPARI2 iNeurons were treated with TTX or various concentrations of glutamate (0, 1, 10, 30, 60, or 100 μM) and illuminated with UV to measure photoconversion of CaMPARI2. Photoconversion increased in a dose-dependent manner.

(b) CaMPARI2 response to glutamate with co-treatment of GluR antagonists and TTX. CaMPARI2 activation is reduced to baseline (neurons treated with 1.5 μM TTX) when treated with APV and NBQX. Activation is not fully reduced to baseline with a full spectrum cocktail of mGluR antagonists, but is still lower than glutamate treatment alone. N = 6 independent culture wells. Source data are provided as a Source Data file.

(c) CaMPARI2 dose response to KCl. CaMPARI2 iNeurons were treated with TTX, 30 μM glutamate, 0.1% DMSO or various concentrations of KCl (10, 30, 50, 100, 300, 500 mM) and illuminated with UV to measure photoconversion of CaMPARI2. Photoconversion increased in a dose-dependent manner to a peak at 50 mM, where a decline in photoconversion was observed at higher concentrations of KCl.

(d) Quantification of CaMPARI2 red/green fluorescence ratio from (b). A non-linear fit could not be established, however the 50 mM KCl treatment was selected for later experiments due to the high conversion and range of response. N = 6 independent culture wells. Source data are provided as a Source Data file.

(e) CaMPARI photoconversion in response to various UV durations. CaMPARI2 iNeurons were treated with TTX or various concentrations of glutamate (0, 1, 10, or 100 μM) and illuminated with UV for various durations of time (0, 1, 5, 10, or 15 min). After illumination, cells were dissociated for flow cytometry and analyzed. N = 5 independent culture wells. Source data are provided as a Source Data file.

(f) Measuring effect of cell density on CaMPARI photoconversion. Significant deviations from optimal plating conditions had no significant effects on photoconversion upon TTX, vehicle, or glutamate treatment. N=4 independent culture wells. Source data are provided as a Source Data file.

(g) Measuring UV toxicity in CaMPARI iNeurons by Countess cell counter. iNeurons were illuminated with UV for 0, 1, 5, 15, or 60 minutes and stained with Trypan Blue viability stain 5 minutes or 24 hours after illumination to assess UV toxicity. N = 3 independent culture wells. Source data are provided as a Source Data file.

(h) Measuring UV toxicity in CaMPARI iNeurons by flow cytometry viability staining. CaMPARI iNeurons were illuminated with UV for 0, 1, 5, 15, or 30 minutes. After illumination, cells were stained TO-PRO-3 for 30 minutes followed by dissociation for analysis by flow cytometry. Percentage viability shows proportion of cells in TO-PRO-3 negative population. N = 9 independent culture wells. Source data are provided as a Source Data file.

(i) Measuring glutamate toxicity. CaMPARI iNeurons were treated with 0, 1, 3, 10, 30, 50, 100 μM of glutamate for 5 minutes and UV illuminated for 5 minutes. TO-PRO-3 staining was performed to measure acute glutamate treatment toxicity as above. N = 3 independent culture wells. Source data are provided as a Source Data file.

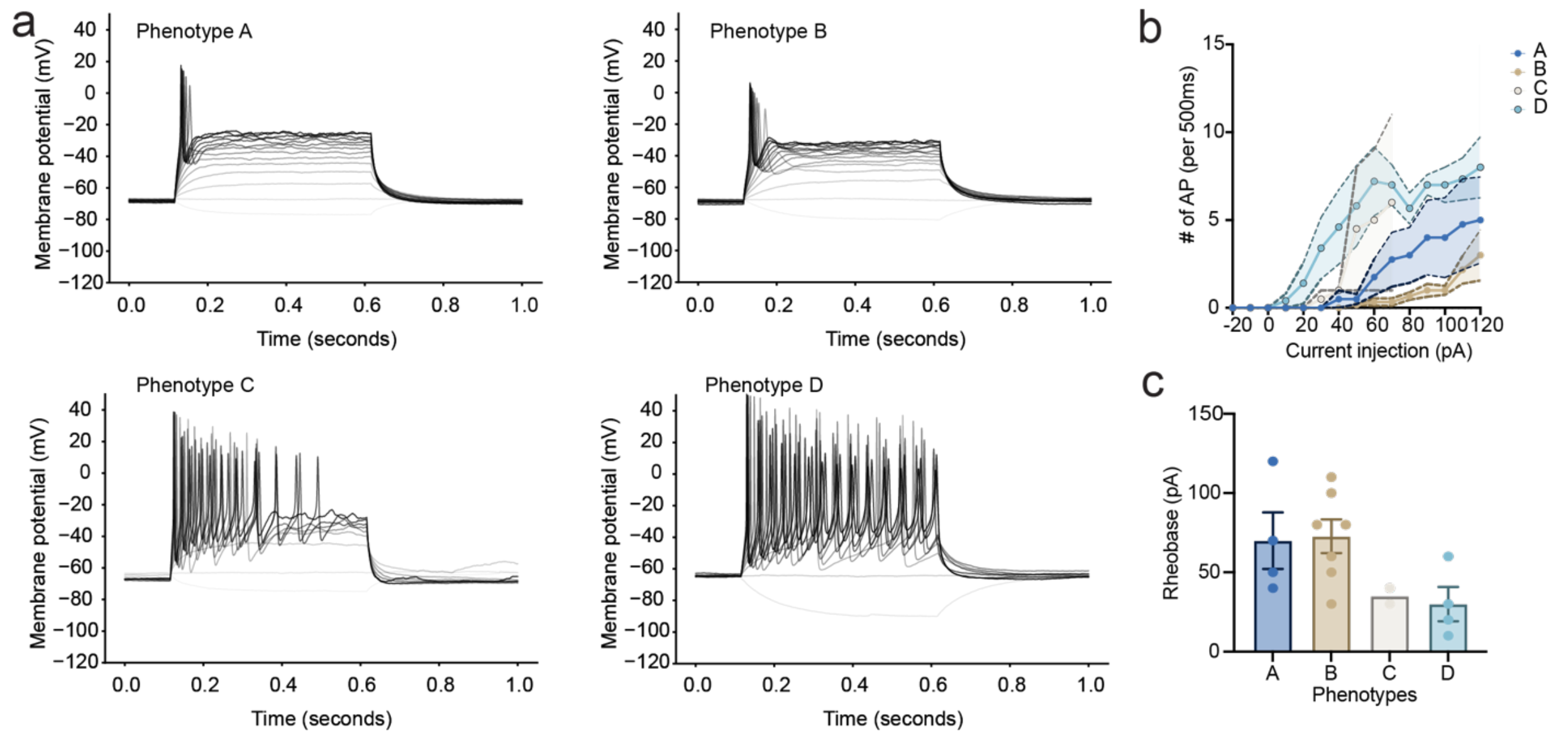

Supplementary Figure 2. iNeurons have heterogeneous responses to glutamate stimulation

(a) Representative voltage responses from the current-spike output recordings of the corresponding iNeurons in Figure 1f-h. Source data are provided as a Source Data file.

(b) Current-spike output relationships for all recorded iNeurons in Figure 1f-h. N = 4 (phenotype A), 6 (phenotype B), 2 (phenotype C), and 5 (phenotype D) independent neurons, 18 neurons total. Shaded error bars are S.E.M. Source data are provided as a Source Data file.

(c) Rheobase values for all recorded iNeurons in Figure 1f-h. N = N = 4 (phenotype A), 6 (phenotype B), 2 (phenotype C), and 5 (phenotype D) independent neurons, 18 neurons total. One-way ANOVA, p = 0.05. Error bars are S.E.M. Source data are provided as a Source Data file.

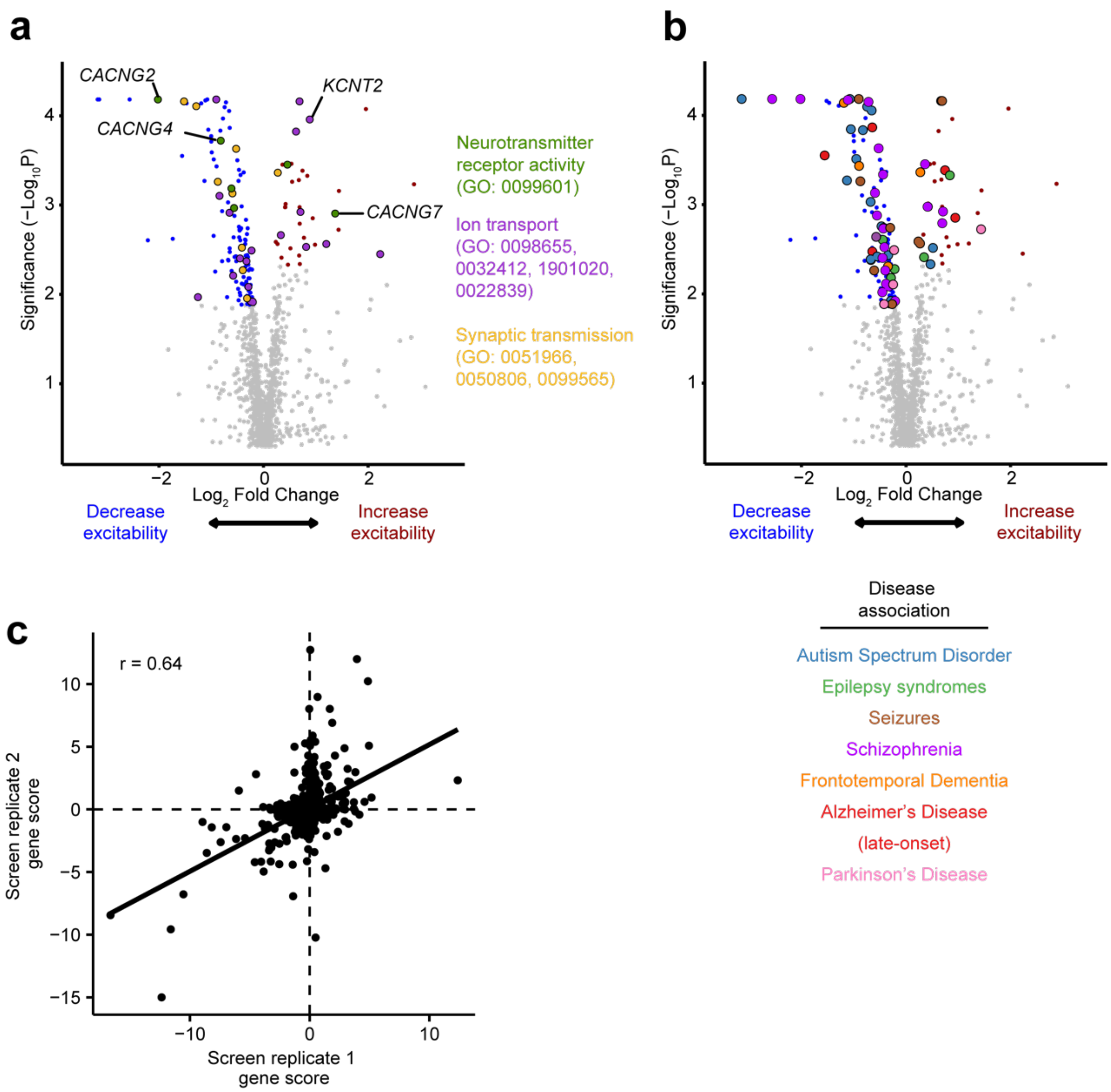
a
CACNG2
CACNG4
KCNT2
CACNG7
Significance (−Log10P)
Log2 Fold Change
Decrease excitability
Increase excitability
Neurotransmitter receptor activity (GO: 0099601)
Ion transport (GO: 0098655, 0032412, 1901020, 0022839)
Synaptic transmission (GO: 0051966, 0050806, 0099565)
b
Significance (−Log10P)
Log2 Fold Change
Decrease excitability
Increase excitability
Disease association
Autism Spectrum Disorder
Epilepsy syndromes
Seizures
Schizophrenia
Frontotemporal Dementia
Alzheimer's Disease (late-onset)
Parkinson's Disease
c
r = 0.64
Screen replicate 2 gene score
Screen replicate 1 gene score

Supplementary Figure 3. Results from Primary screen

(a) Volcano plot summarizing the effect of knockdowns on CaMPARI phenotypes and the determine statistical significance for targeted genes (Mann-Whitney U test). Dashed lines indicate a false-discovery rate (FDR) cutoff of 0.05, based on the phenotype score for gene calculated from 5 targeting sgRNAs. Black points indicate hit genes where knockdown changes CaMPARI red/green fluorescence ratio in response to glutamate stimulation, while grey points indicate non-hits. Genes related to synaptic transmission (purple) and ion transport (orange) are highlighted based on Gene Ontology terms. Source data are provided as a Source Data file.

(b) Disease associated genes from DisGenNet and whole-exome sequencing (Satterstrom et al)[1] overlap with hit genes from CaMPARI screen. Source data are provided as a Source Data file.

(c) Scatterplot of gene scores from each replicate of the primary screen shown the correlation between screens. Pearson's R = 0.49, linear regression fit depicted by solid black line. Source data are provided as a Source Data file.

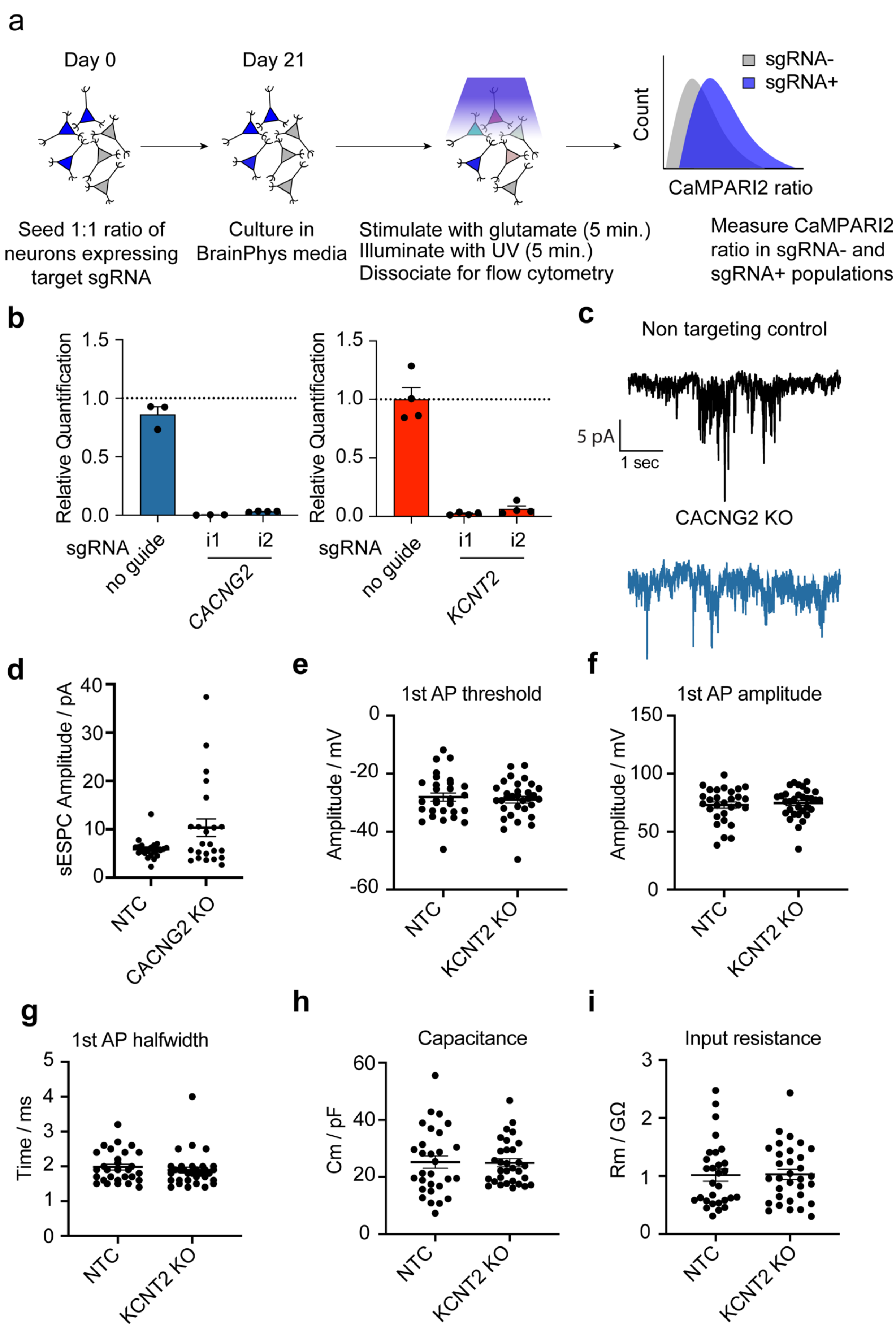
a
Day 0
Day 21
Count
sgRNA-
sgRNA+
CaMPARI2 ratio
Seed 1:1 ratio of neurons expressing target sgRNA
Culture in BrainPhys media
Stimulate with glutamate (5 min.)
Illuminate with UV (5 min.)
Dissociate for flow cytometry
Measure CaMPARI2 ratio in sgRNA- and sgRNA+ populations
b
Relative Quantification
sgRNA
no guide
i1
i2
CACNG2
KCNT2
c
Non targeting control
5 pA
1 sec
CACNG2 KO
d
sESPC Amplitude / pA
NTC
CACNG2 KO
e
1st AP threshold
Amplitude / mV
KCNT2 KO
f
1st AP amplitude
g
1st AP halfwidth
Time / ms
h
Capacitance
Cm / pF
i
Input resistance
Rm / GΩ

Supplementary Figure 4. Electrophysiology profiles of iNeurons with KCNT2 and CACNG2 knockdowns.

(a) Schematic of intra-well comparison experiments with 50% of CaMPARI2 iNeurons expressing a sgRNA for a target gene. A 1:1 ratio of iNeurons expressing the sgRNA: not expressing a sgRNA are seeded and differentiated. These are then treated with the same glutamate and photoconversion conditions. These are gated based on the presence of BFP in the sgRNA construct, and the CaMPARI2 ratio is directly compared between the two populations.

(b) RT-qPCR shows the knock down efficiency of the sgRNAs targeting CACNG2 (blue) and KCNT2 (red). N = 4 biological replicates per condition. Error bars are SEM. Source data are provided as a Source Data file.

(c) Sample sEPSC recordings from NTC (black trace) and CACNG2 KO (blue trace) iNeurons. Source data are provided as a Source Data file.

(d) CACNG2 KO iNeurons show a slight increase in sEPSC amplitude that is not statistically significant. (NTC: 22 neurons, CACNG2 KO:23 neurons, Mann-Whitney test. Source data are provided as a Source Data file.

(e-i) No differences between NTCs (N= 29 neurons) and KNCT2 KOs (N = 32 neurons) in either passive membrane properties or action potential kinetics. Error bars donate standard error. Source data are provided as a Source Data file.

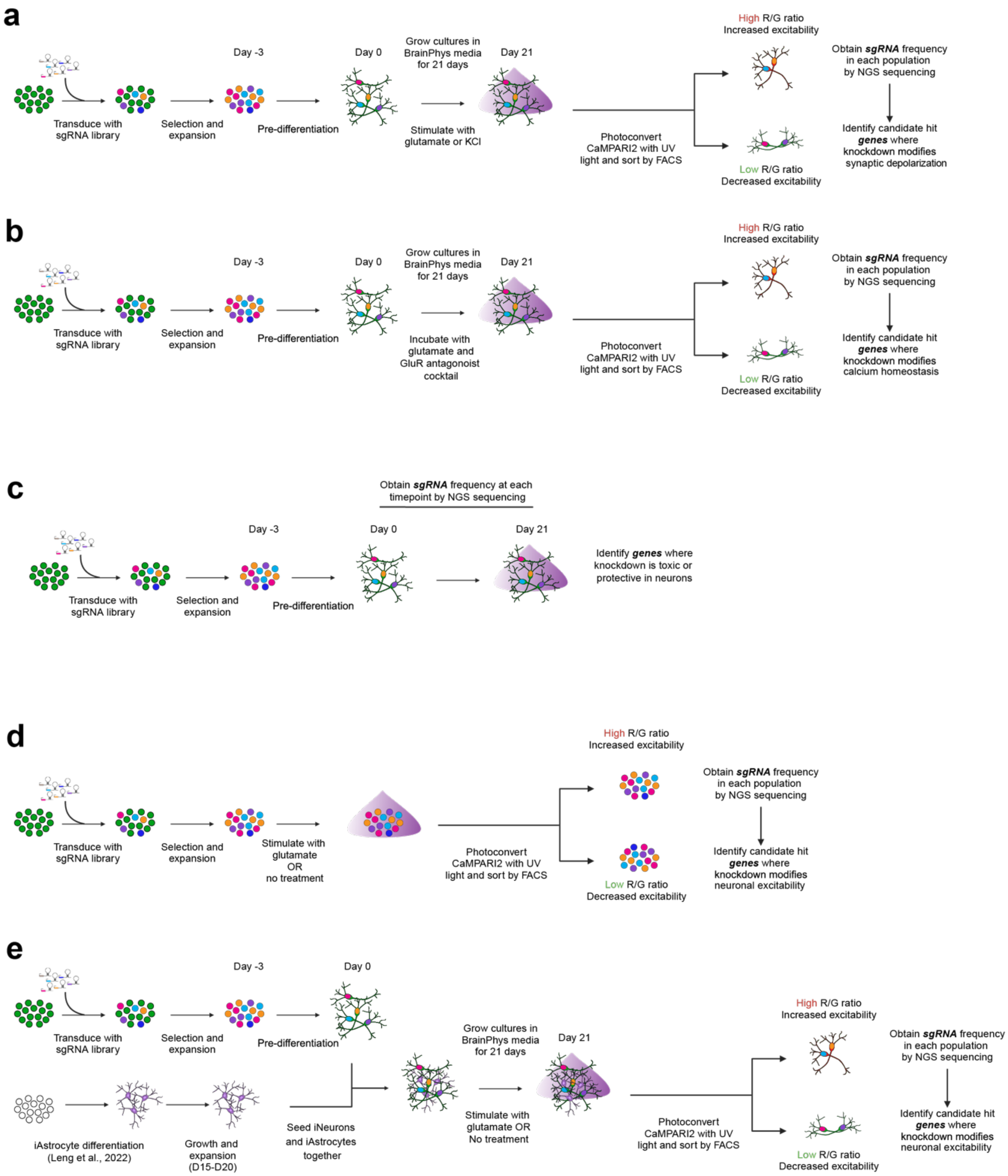
a
Transduce with sgRNA library
Selection and expansion
Day -3
Pre-differentiation
Day 0
Grow cultures in BrainPhys media for 21 days
Stimulate with glutamate or KCl
Day 21
Photoconvert CaMPARI2 with UV light and sort by FACS
High R/G ratio Increased excitability
Low R/G ratio Decreased excitability
Obtain sgRNA frequency in each population by NGS sequencing
Identify candidate hit genes where knockdown modifies synaptic depolarization
b
Transduce with sgRNA library
Selection and expansion
Day -3
Pre-differentiation
Day 0
Grow cultures in BrainPhys media for 21 days
Incubate with glutamate and GluR antagonoist cocktail
Day 21
Photoconvert CaMPARI2 with UV light and sort by FACS
High R/G ratio Increased excitability
Low R/G ratio Decreased excitability
Obtain sgRNA frequency in each population by NGS sequencing
Identify candidate hit genes where knockdown modifies calcium homeostasis
c
Obtain sgRNA frequency at each timepoint by NGS sequencing
Transduce with sgRNA library
Selection and expansion
Day -3
Pre-differentiation
Day 0
Day 21
Identify genes where knockdown is toxic or protective in neurons
d
Transduce with sgRNA library
Selection and expansion
Stimulate with glutamate OR no treatment
Photoconvert CaMPARI2 with UV light and sort by FACS
High R/G ratio Increased excitability
Low R/G ratio Decreased excitability
Obtain sgRNA frequency in each population by NGS sequencing
Identify candidate hit genes where knockdown modifies neuronal excitability
e
Transduce with sgRNA library
Selection and expansion
Day -3
Pre-differentiation
Day 0
iAstrocyte differentiation (Leng et al., 2022)
Growth and expansion (D15-D20)
Seed iNeurons and iAstrocytes together
Grow cultures in BrainPhys media for 21 days
Stimulate with glutamate OR No treatment
Day 21
Photoconvert CaMPARI2 with UV light and sort by FACS
High R/G ratio Increased excitability
Low R/G ratio Decreased excitability
Obtain sgRNA frequency in each population by NGS sequencing
Identify candidate hit genes where knockdown modifies neuronal excitability

Supplementary Figure 5. Secondary CRISPRi screen strategies.

(a) Screening strategy. CRISPRi iPSCs were transduced with a pooled lentivirus sgRNA library consisting of hit genes from the primary screen (1622 sgRNAs targeting 424 genes). Exposure to puromycin selects for iPSCs successfully transduced with sgRNA construct. iPSCs were then differentiated into neurons via overexpression of NGN2 and cultured for 21 days. Neurons were then incubated with either 30 μM glutamate or 50 mM KCl, illuminated with UV light, and then separated by FACS into populations with high versus low red/green fluorescence ratios.

(b) iPSCs were differentiated into neurons via overexpression of NGN2 and cultured for 21 days. Neurons were then incubated with 30 μM glutamate, 1.5 μM TTX, 10 μM NBQX, 50 μM APV, 100 μM LY367385, 0.1 μM LY341495, 1 μM CPPG, 1 μM MTEP. Neurons were then illuminated with UV light, and then separated by FACS into populations with high versus low red/green fluorescence ratios.

(c) Survival of neurons was assessed by comparing sgRNA frequency in neurons at the start of differentiation (day 0) and after 21 days of differentiation.

(d) iPSCs expressing sgRNAs were treated with 30 μM glutamate for 5 minutes, illuminated with UV light, and then separated by FACS into populations with high versus low red/green fluorescence ratios.

(e) iPSCs expressing sgRNAs were differentiated into neurons and combined with Day 20 iAstrocytes. These co-cultures were grown for 21 days. Neurons were then treated 30 μM glutamate, illuminated with UV light, and then separated by FACS into populations with high versus low red/green fluorescence ratios. To assess the spontaneous activity of these cultures, no glutamate stimulation was applied.

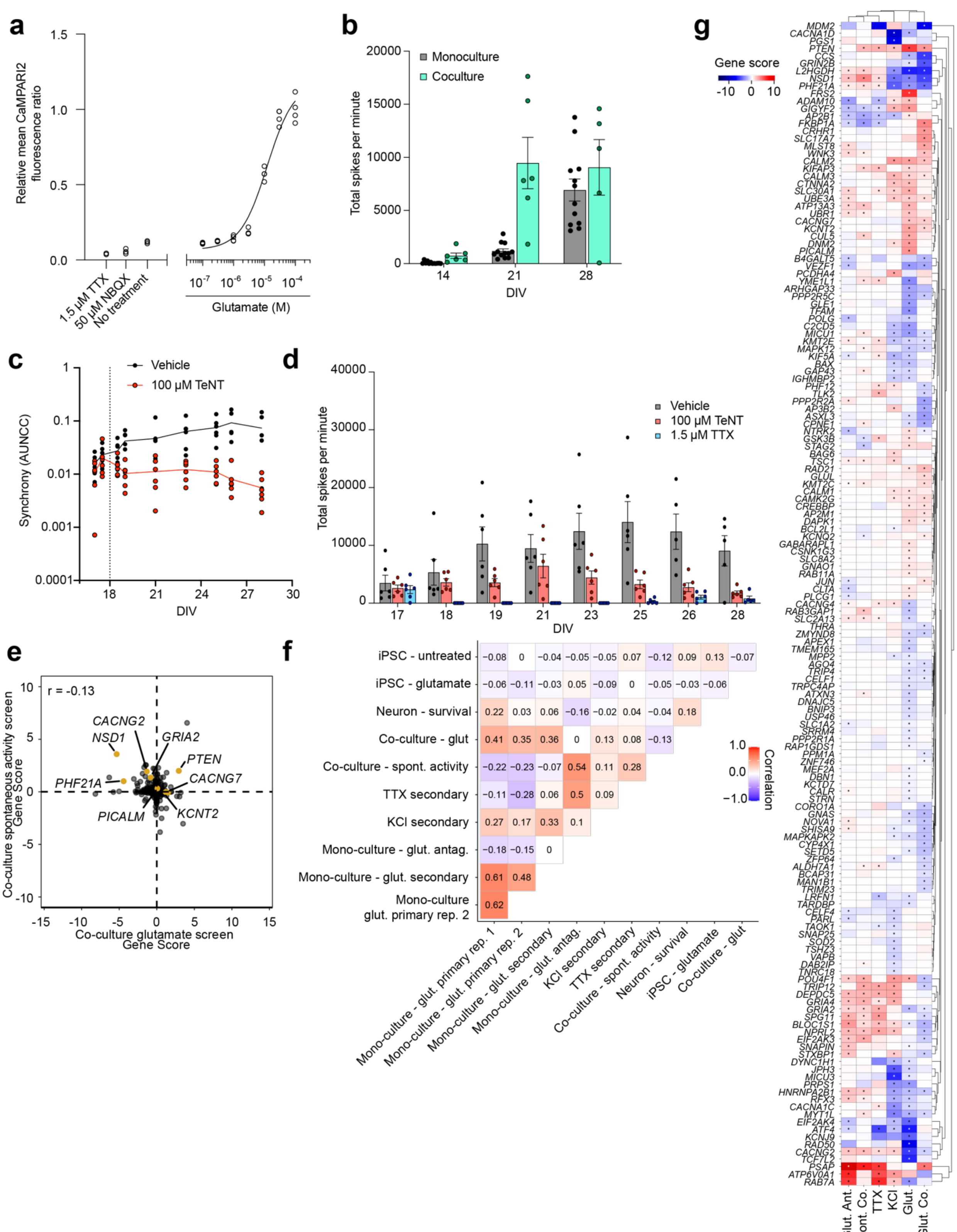

a
Relative mean CaMPARI2 fluorescence ratio
1.5 µM TTX
50 µM NBQX
No treatment
Glutamate (M)
b
Monoculture
Coculture
Total spikes per minute
DIV
c
Vehicle
100 µM TeNT
Synchrony (AUNCC)
DIV
d
Vehicle
100 µM TeNT
1.5 µM TTX
Total spikes per minute
DIV
e
r = -0.13
CACNG2
NSD1
GRIA2
PTEN
CACNG7
PHF21A
PICALM
KCNT2
Co-culture spontaneous activity screen Gene Score
Co-culture glutamate screen Gene Score
f
iPSC - untreated -0.08 0 -0.04 -0.05 -0.05 0.07 -0.12 0.09 0.13 -0.07
iPSC - glutamate -0.06 -0.11 -0.03 0.05 -0.09 0 -0.05 -0.03 -0.06
Neuron - survival 0.22 0.03 0.06 -0.16 -0.02 0.04 -0.04 0.18
Co-culture - glut 0.41 0.35 0.36 0 0.13 0.08 -0.13
Co-culture - spont. activity -0.22 -0.23 -0.07 0.54 0.11 0.28
TTX secondary -0.11 -0.28 0.06 0.5 0.09
KCl secondary 0.27 0.17 0.33 0.1
Mono-culture - glut. antag. -0.18 -0.15 0
Mono-culture - glut. secondary 0.61 0.48
Mono-culture glut. primary rep. 2 0.62
Mono-culture - glut. primary rep. 1
Mono-culture - glut. primary rep. 2
Mono-culture - glut. secondary
Mono-culture - glut. antag.
KCl secondary
TTX secondary
Co-culture - spont. activity
Neuron - survival
iPSC - glutamate
Co-culture - glut
Correlation
g
Gene score
Glut. Ant.
Spont. Co.
TTX
KCl
Glut.
Glut. Co.

Supplementary Figure 6. Secondary CRISPRi screens reveal excitability phenotypes in iPSC neurons.

(a) CaMPARI2 neurons in coculture with iAstrocytes respond to glutamate in a dose-dependent manner. N = 4 independent culture wells per measurement. TTX (1.5 μM), NBQX (50 μM), and "no treatment" conditions did not contain glutamate. Source data are provided as a Source Data file.

(b) Multielectrode arrays (MEA) recordings of spikes per minute of neuron-astrocyte cocultures and monoculture neurons. After 21 days of culture, more spikes are detected in cocultures. N = 6 independent culture wells, each with 16 electrodes. Source data are provided as a Source Data file.

(c) Synchrony of neuronal activity in neuron-astrocyte cocultures on MEA as measured through Area Under a Normalized Cross Correlation. After recording on day 18 (dotted line), cultures were treated with 100 μM tetanus toxin (TeNT) or vehicle control. Loss of synchrony begins around 24 hours later in TeNT treated wells, while vehicle treated wells show a slight increase. N = 6 independent culture wells, with 16 electrodes per well. Source data are provided as a Source Data file.

(d) Total spikes per minute of cultures from (c). Day 18 recording measured before addition of TeNT. N = 6 independent culture wells per condition, with 16 electrodes per well. Source data are provided as a Source Data file.

(e) Modifiers of activity of spontaneously firing neurons in coculture do not correlate with those of glutamate-stimulated neurons in coculture (Pearson's R = -0.13). Source data are provided as a Source Data file.

(f) Correlation matrix of gene scores from CaMPARI2 screens, using Pearson's correlation R.

(g) Heatmap of gene scores in secondary screens. Each included gene is classified as a hit in at either the KCl or glutamate (monoculture and coculture) screens (FDR > 0.1) and is denoted with an asterisk when classified as a hit.

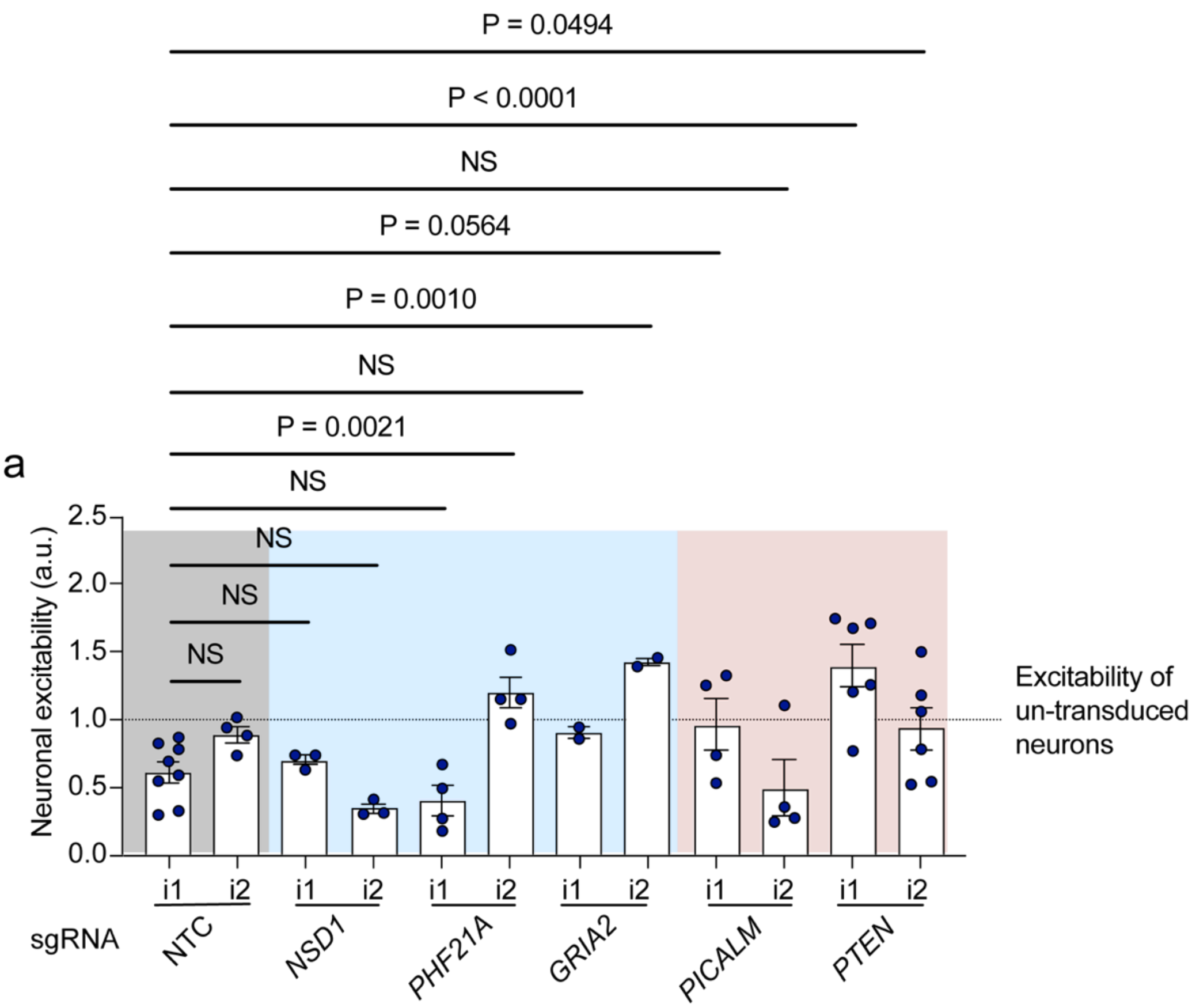

Supplementary Figure 7. Validation of KDs from secondary screens

In cultures where 100% of CaMPARI2 iNeurons are expressing sgRNAs targeting NSD1 and PHF21A, these iNeurons lack statistically significant reduced neuronal excitability in response to glutamate stimulation observed in the screen relative to non-targeting controls. iNeurons expressing sgRNAs targeting PTEN show the expected increase in activity. For iNeurons expressing sgRNAs targeting GRIA2 and PICALM, the expected screen phenotype is not preserved when 100% of the cells have the knockdown. CaMPARI2 ratios are normalized to neuron samples that do not receive a sgRNA and are run on the flow cytometer on the same day. N = 8 (NTC_i1), 3 (NTC_i2), 2 (NSD1_i1), 2 (NSD1_i2), 3 (PHF21A_i1), 3 (PHF21A_i2), 2 (GRIA2_i1), 2 (GRIA2_i2), 4 (PICALM_i1), 4 (PICALM_i2), 6 (PTEN_i1), 6 (PTEN_i2) independent culture wells. Each individual experiment was normalized to the excitability (CaMPARI2 response) of wells where neurons did not receive a sgRNA. Error bars denote standard error. P values calculated by ordinary one-way ANOVA, using NTC_i1 as reference. P > 0.05 = not significant (NS). Source data are provided as a Source Data file.

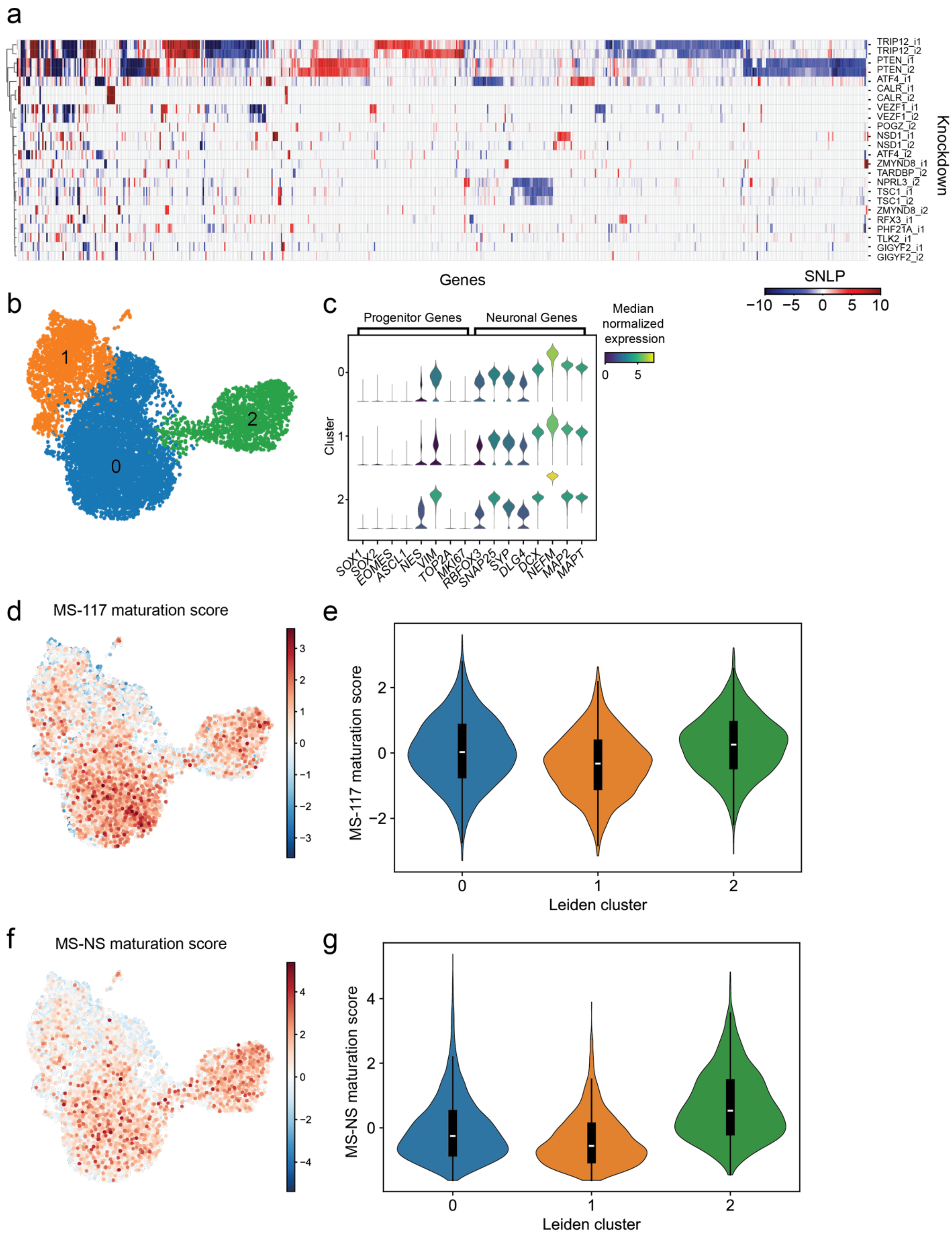
a
TRIP12_i1
TRIP12_i2
PTEN_i1
PTEN_i2
ATF4_i1
CALR_i1
CALR_i2
VEZF1_i1
VEZF1_i2
POGZ_i2
NSD1_i1
NSD1_i2
ATF4_i2
ZMYND8_i1
TARDBP_i2
NPRL3_i2
TSC1_i1
TSC1_i2
ZMYND8_i2
RFX3_i1
PHF21A_i1
TLK2_i1
GIGYF2_i1
GIGYF2_i2
Knockdown
Genes
SNLP
−10 −5 0 5 10
b
c
Progenitor Genes
Neuronal Genes
Median normalized expression
Cluster
SOX1 SOX2 EOMES ASCL1 NES VIM TOP2A MKI67 RBFOX3 SNAP25 SYP DLG4 DCX NEFM MAP2 MAPT
d
MS-117 maturation score
e
MS-117 maturation score
Leiden cluster
f
MS-NS maturation score
g
MS-NS maturation score
Leiden cluster

Supplementary Figure 8. CROP-seq reveals transcriptional changes to genes involved in synaptic processes drive changes in excitability.

(a) Heatmap of differentially expressed genes (DEGs, versus non-targeting controls, adjusted p-value < 0.05) for knockdowns with significant on-target KD. Clustering shows consistency between individual sgRNAs targeting the same gene. SNLP is signed negative log of the adjusted p-value. Knockdowns with >10 DEGs are shown.

(b) Leiden clustering of neurons from CROP-seq classified into 3 clusters.

(c) Marker gene expression is homogenous across clusters and suggests that neuronal identity is conserved.

(d) MS-117 score[2] based on expression of 117 maturation marker genes.

(e) Violin plot of MS-117 score grouped by leiden clusters.

(f) MS-NS score[2] based of expression of 25 neuron-specific marker genes.

(g) Violin plot of MS-NS score shows slight increase in maturation score in cluster 2.

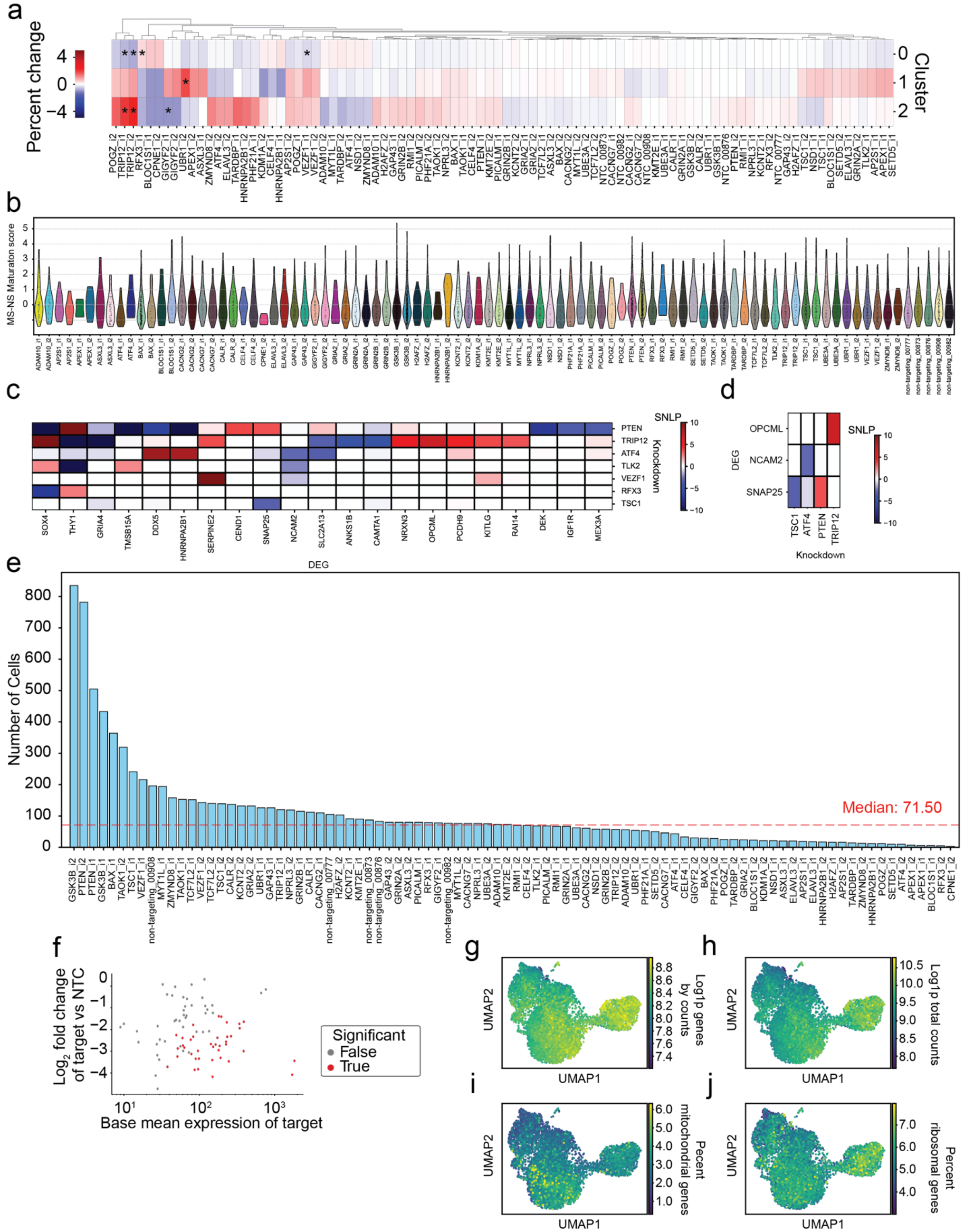

a
Percent change
Cluster
b
MS-NS Maturaton score
c
SNLP
Knockdown
DEG
PTEN
TRIP12
ATF4
TLK2
VEZF1
RFX3
TSC1
SOX4
THY1
GRIA4
TMSB15A
DDX5
HNRNPA2B1
SERPINE2
CEND1
SNAP25
NCAM2
SLC2A13
ANKS1B
CAMTA1
NRXN3
OPCML
PCDH9
KITLG
RAI14
DEK
IGF1R
MEX3A
d
OPCML
NCAM2
SNAP25
DEG
SNLP
Knockdown
e
Number of Cells
Median: 71.50
f
Log$_2$ fold change of target vs NTC
Base mean expression of target
Significant
False
True
g
UMAP2
UMAP1
Log1p genes by counts
h
UMAP2
UMAP1
Log1p total counts
i
UMAP2
UMAP1
Pecent mitochondrial genes
j
UMAP2
UMAP1
Percent ribosomal genes

Supplementary Figure 9. CROP-seq reveals transcriptional changes to genes involved in synaptic processes drive changes in excitability.

(a) Heatmap of cluster shift analysis for each knockdown. Color values are expressed as the percent change in abundance of the knockdown in each cluster relative to non-targeting controls. Asterisks denote significant enrichments and depletions (adjusted q-value < 0.05).

(b) Violin plot of MS-NS score for each cell grouped by sgRNA assignment.

(c) Heatmap of MS-117 maturation marker genes[2] that are significant DEGs upon knockdown of target genes. SNLP is signed negative log of the adjusted p-value.

(d) Heatmap of MS-NS maturation marker genes[68] that are significant DEGs upon knockdown of target genes. SNLP is signed negative log of the adjusted p-value.

(e) sgRNA representation by number of neurons detected in scRNA-seq data post quality control filtering and with a sgRNA MOI of one.

(f) Plot of on-target KD versus base mean expression for each sgRNA. Lower expression of genes generally resulted in less ability to call significance.

(g) Log(1+P) genes detected in each cell.

(h) Log(1+P) total read counts in each cell.

(i) Percentage of mitochondrial genes detected in each cell.

(j) Percentage of ribosomal genes detected in each cell.

Supplementary Table 1. Primers used for qPCR of sgRNA targets

Target	5' sequence (forward)	3' sequence (reverse)
NSD1	CTTTTCCAAGCGCCAATATCC	GCTCACCCACATACTCATTCAC
PHF21A	TCATTTGGACTGCTTAGACCC	CAATTGCTAAAGTTCCAGGCC
PICALM	GCAATGAACTTACAAATGGGGT	AAGACCTTCTTTGCATTGGTTC
PTEN	TCACCAACTGAAGTGGCTAAAGA	CTCCATTCCCCTAACCCGA
KCNT2	TTGCCAGCATGGGTACTGTG	AGGAAGAGACAGGGTAGGGC
CACNG2	GACCAAAAGTGTCAGTGAGAATG	CTTCGTAATCTGCATCCTCTGG
GAPDH	ATGCCTCCTGCACCACCAAC	GGGGCCATCCACAGTCTTCT

Supplementary Table 2. sgRNA sequences used to generate KOs for electrophysiogical characterization

Target	sgRNA1	sgRNA2	sgRNA3
PICALM	CUUGCAUACUGUCUUGGAUA	UCGGUCCGUCAGGCUCUGGC	GUCGGCUUCACUCACAGUCC
CACNG2	GGUUCUCCAUAAUCCGGAAU	UUUGGUCUUGCAAACCCCUC	GGCAGCGAAAGCACCAACGG
KCNT2	AUACCUACUUUGCUCUCAAU	AUAACUCGUUGGGACCACAU	UUUUGAAUAGGAUUAUUAUG

Supplementary Table 3. sgRNA protospacer sequences used for KD validation experiments

Gene	Guide ID	Guide name	Protospacer Sequence
PICALM	PICALM_-_85779699.23-P1P2	PICALM_i1	GTCGGCTTCACTCACAGTCC
PICALM	PICALM_-_85780018.23-P1P2	PICALM_i2	GCTCGTGTCACCCGCGGAGT
GRIA2	GRIA2_-_158141995.23-P1P2	GRIA2_i1	GGACTTCTGGAGCGGGGACA
GRIA2	GRIA2_+_158141968.23-P1P2	GRIA2_i2	GCCCCGCTCCAGAAGTCCGG
NSD1	NSD1_+_176561172.23-P1P2	NSD1_i1	GCGCGCCGGGAGGAAGTTCG
NSD1	NSD1_+_176561201.23-P1P2	NSD1_i2	GTGTTCGCAGGCGGCCGGCG
CACNG2	CACNG2_+_37099513.23-P1P2	CACNG2_i1	GCCAGCCGGTAGCGAGTGGG
CACNG2	CACNG2_+_37099516.23-P1P2	CACNG2_i2	GCGCCCAGCCGGTAGCGAGT
NTC	non-targeting_00777	NTC_i2	GGCTCGTTGGACAAACGTAG
NTC	non-targeting_00089	NTC_i1	GGGGTGAGGGTCCAATTCGG
NTC	non-targeting_00876	NTC_i3	GTCGGACCAAGCAGGCAGAA
KCNT2	KCNT2_-_196577542.23-P1P2	KCNT2_i1	GGAGGACTAACAAGACGCTG
KCNT2	KCNT2_-_196577553.23-P1P2	KCNT2_i2	GAGACGCTGTGGCCGAGAGA
CACNG7	CACNG7_-_54412952.23-P1P2	CACNG7_i1	GCTGGGCCTGGCGATCCCGG
CACNG7	CACNG7_-_54413017.23-P1P2	CACNG7_i2	GTCCGAGTCGCAGCGAGCGT
PHF21A	PHF21A_+_46142971.23-P1P2	PHF21A_i1	GGGCCGGGTGGTGAATGGGC
PHF21A	PHF21A_-_46142369.23-P1P2	PHF21A_i2	GACACCAGCGACAAGACAGA
PTEN	PTEN_-_89623395.23-P1P2	PTEN_i1	GGCGCTCAGTTCTCTCCTCT
PTEN	PTEN_+_89623582.23-P1P2	PTEN_i2	GGTTAGAAAAGACGAAGAGG

Citations:

1. Satterstrom, F. K. *et al.* Large-Scale Exome Sequencing Study Implicates Both Developmental and Functional Changes in the Neurobiology of Autism. *Cell* **180**, 568-584.e23 (2020).
2. Shan, X. *et al.* Fully defined NGN2 neuron protocol reveals diverse signatures of neuronal maturation. *Cell Rep. Methods* **4**, 100858 (2024).
