# Supplementary material for: A massively parallel CRISPR-based screening platform for modifiers of neuronal depolarization
Source: Nat Commun. 2026 Aug 1;17:9299. doi: 10.1038/s41467-026-75882-0 (PMC13526836; doi:10.1038/s41467-026-75882-0)
Supplement: Supplementary file 3 [file 41467_2026_75882_MOESM2_ESM.pdf]

nature portfolio

Peer Review File

A Massively Parallel CRISPR-Based Screening Platform for Modifiers of Neuronal Depolarization

Corresponding Author: Dr Martin Kampmann

This file contains all reviewer reports in order by version, followed by all author rebuttals in order by version.

This manuscript has been previously reviewed at another journal. This document only contains information relating to versions considered at Nature Communications.

Version 0:

Reviewer comments:

Reviewer #2

(Remarks to the Author)
The revised manuscript is substantially improved and ready for publication. Most comments are addressed experimentally. In addition, text has been modified to provide more explanations about the experiments and results. The only minor concern that the authors should address is to revise the text based on their response to the reviewer's comment below

Reviewer #2: "Validity of CAMPARI signals: how do the authors explain the CAMPARI signal in Ext Fig 1b (+Glu +TTX leads to essentially no difference than +Glu alone)? If TTX ablates the transmission
(supported by Ext Fig 6), what are the signals that CAMPARI measures? Are they relevant?

Authors response: "TTX blocks the propagation of action potentials through a Na channel block, so in the context of the +Glu+TTX condition, the Campari signal measures the depolarization and calcium response from activated glutamate receptors of each individual neuron. The +Glu +TTX condition in Extended data figure 1b only shows a slight, likely insignificant decrease in the CaMPARI response. This strongly suggests that most of the CaMPARI response triggered by glutamate stimulation is likely through cell-autonomous depolarization via glutamate receptors"

Comment: Based on this explanation, the authors should consider modifying the title and abstract. It appears the genes identified in the screen are related to neuronal depolarization, not to "Neuronal Activity," as stated in the title and abstract.

The manuscript should also be proofread for typos. Here are a few that the reviewer identified:

"Here, we CaMPARI2 in human iPSC-derived neurons"

"Glutamate antagonism screen"

Reviewer #4

(Remarks to the Author)
This manuscript presents an interesting genetic screen that combines CRISPRi-mediated gene perturbation with the fluorescent calcium integrator CaMPARI2. The authors apply this approach to human iPSC-derived neurons and screen over 1,000 genes implicated in activity regulation or brain disease. By quantifying gRNA frequencies in neurons with altered excitability (based on the CaMPARI2 red/green ratio, comparing the top 35% and bottom 35% bins), they identify candidate genes that modulate neuronal activity in vitro. While this approach yields valuable insights, neuronal activity in cultured iPSC-derived neurons differs substantially from the physiological context in vivo. Demonstrating the applicability of this tool in more physiologically relevant systems (e.g., cerebral organoids) would greatly strengthen the manuscript.
Major:
1. Concern regarding cell health. Although the authors indicate that 5 minutes of UV illumination had minimal immediate effects on cell viability, Extended Data Figure 1g shows that live cell numbers decreased to ~10% at 24 hours post-UV. In contrast, Extended Data Figure 1h appears to show much better viability. Could the authors clarify at what time point the viability was assessed in Extended Data Figure 1h, and explain the discrepancy between these two datasets? Furthermore, I recommend that the authors evaluate cell viability over longer time points (e.g., 1 day and 7 days post-UV) to ensure that the neuronal cultures remain healthy throughout the assay.

2. Concern regarding neuronal maturity. 1) In the current ephys dataset, only 7 out of 18 neurons responded to 30 µM glutamate treatment, suggesting that over half of the recorded neurons may be immature. If this is the case, it would be more appropriate to apply the tool in a more mature neuronal system rather than relying on iNeurons that exhibit limited neuronal activity. 2) Furthermore, a sample size of 18 neurons is insufficient to draw strong conclusions. The authors should provide ephys data from a larger number of neurons across multiple culture batches. It would also be informative to examine whether a higher concentration of glutamate (e.g., 100 µM) elicits similar or distinct response profiles in iNeurons. 3) Since the majority of ASD risk genes influence neurodevelopment, using iNeurons, which are relatively immature, as the model may overlook important gene functions related to neuronal maturation.
3. Include the co-culture system in the primary screen. The co-culture condition produced distinct outcomes compared to the monoculture condition, showing low correlation with monocultured neurons(Fig. 4) and different phenotypes of genetic perturbations (Line 340-346). Given these differences, it is important to include co-cultured neurons in the primary screen to capture the full spectrum of gene function. In addition, it would be great to assess the toxicity of UV and glutamate in the co-cultured system.
4. CROP-seq data confusion. 1) The authors should identify the cell clusters present in their dataset. It is confusing that they stated neuronal differentiation was not a major contributor to the observed changes, yet cannot distinguish different differentiation stages within their iNeuron cultures. 2) Furthermore, the median cell number per perturbation is only 71.5, which is insufficient for robust analysis and limits the strength of the conclusions. Additional data with increased coverage per perturbation will be necessary.
Minor:
1. In Fig. 2b, the dashed line is missing.
2. In Line 210, the FDR should be consistent with the figure legend.

Version 1:

Reviewer comments:

Reviewer #4

(Remarks to the Author)
I am supportive of publication. I appreciate the effort the authors have made to address the previous comments and to incorporate these points into the discussion.
I still note that the generalizability of the technology is mainly limited to the current in vitro system and is not demonstrated beyond this context. This doesn't prevent publication, but it's something to keep in mind when considering the broader applicability.

Open Access This Peer Review File is licensed under a Creative Commons Attribution 4.0 International License, which permits use, sharing, adaptation, distribution and reproduction in any medium or format, as long as you give appropriate credit to the original author(s) and the source, provide a link to the Creative Commons license, and indicate if changes were made.
In cases where reviewers are anonymous, credit should be given to 'Anonymous Referee' and the source.
The images or other third party material in this Peer Review File are included in the article's Creative Commons license,

unless indicated otherwise in a credit line to the material. If material is not included in the article's Creative Commons license and your intended use is not permitted by statutory regulation or exceeds the permitted use, you will need to obtain permission directly from the copyright holder.
To view a copy of this license, visit https://creativecommons.org/licenses/by/4.0/

Response to reviewer comments

Reviewer #2 (Remarks to the Author):

The revised manuscript is substantially improved and ready for publication. Most comments are addressed experimentally. In addition, text has been modified to provide more explanations about the experiments and results. The only minor concern that the authors should address is to revise the text based on their response to the reviewer's comment below

Reviewer #2: "Validity of CAMPARI signals: how do the authors explain the CAMPARI signal in Ext Fig 1b (+Glu +TTX leads to essentially no difference than +Glu alone)? If TTX ablates the transmission (supported by Ext Fig 6), what are the signals that CAMPARI measures? Are they relevant?

Authors response: "TTX blocks the propagation of action potentials through a Na channel block, so in the context of the +Glu+TTX condition, the Campari signal measures the depolarization and calcium response from activated glutamate receptors of each individual neuron. The +Glu +TTX condition in Extended data figure 1b only shows a slight, likely insignificant decrease in the CaMPARI response. This strongly suggests that most of the CaMPARI response triggered by glutamate stimulation is likely through cell-autonomous depolarization via glutamate receptors"

Comment: Based on this explanation, the authors should consider modifying the title and abstract. It appears the genes identified in the screen are related to neuronal depolarization, not to "Neuronal Activity," as stated in the title and abstract.

We agree with the reviewer, and have modified the title, abstract, and other parts in the manuscript to address that the screens in this manuscript reflect neuronal depolarization. Still, we believe this platform could be developed further to directly study mechanisms of neuronal activity.

The manuscript should also be proofread for typos. Here are a few that the reviewer identified:

"Here, we CaMPARI2 in human iPSC-derived neurons"

"Glutamate antagonism screen"

We have carefully proofread the manuscript and addressed the typos pointed out by the reviewer.

Reviewer #4 (Remarks to the Author):

This manuscript presents an interesting genetic screen that combines CRISPRi-mediated gene perturbation with the fluorescent calcium integrator CaMPARI2. The authors apply this approach to human iPSC-derived neurons and screen over 1,000 genes implicated in activity regulation or brain disease. By quantifying gRNA frequencies in neurons with altered excitability (based on the CaMPARI2 red/green ratio, comparing the top 35% and bottom 35% bins), they identify candidate genes that modulate neuronal activity in vitro. While this approach yields valuable insights, neuronal activity in cultured iPSC-derived neurons differs substantially from the physiological context in vivo. Demonstrating the applicability of this tool in more physiologically relevant systems (e.g., cerebral organoids) would greatly strengthen the manuscript.

Major:

1. Concern regarding cell health. Although the authors indicate that 5 minutes of UV illumination had minimal immediate effects on cell viability, Extended Data Figure 1g shows that live cell numbers decreased to ~10% at 24 hours post-UV. In contrast, Extended Data Figure 1h appears to show much better viability. Could the authors clarify at what time point the viability was assessed in Extended Data Figure 1h, and explain the discrepancy between these two datasets? Furthermore, I recommend that the authors evaluate cell viability over longer time points (e.g., 1 day and 7 days post-UV) to ensure that the neuronal cultures remain healthy throughout the assay.

Thank you for pointing out that the two experiments with different assessments of cell viability after UV treatment can cause confusion.

Extended Data Figure 1g is based on a Trypan Blue stain using Countess cell counter to assess the percent viable cells after UV illumination. Indeed, we see a stark decrease in cell viability 24 hours after illumination. However, when cells were processed and analyzed 5 minutes after UV illumination there was little difference in viability up to 5 minutes of UV illumination compared to no UV illumination. Given that this was a fairly crude assessment of viability, we also performed an orthogonal experiment using flow cytometry in cells that were also transduced with non-targeting control guides (Extended Data Figure 1h). Using TO-PRO-3 to mark dead cells, we found that there was little effect on cell viability if cells were dissociated and analyzed 5 minutes after UV illumination was complete. This more closely resembles our screening workflow, thus

we concluded that as long as we proceeded to FACS immediately after UV illumination we could avoid confounding effect of cell death.

We have clarified this point in the revised manuscript in the Figure legend of Extended Data Figure 1, and in the main text.

2. Concern regarding neuronal maturity. 1) In the current ephys dataset, only 7 out of 18 neurons responded to 30 µM glutamate treatment, suggesting that over half of the recorded neurons may be immature. If this is the case, it would be more appropriate to apply the tool in a more mature neuronal system rather than relying on iNeurons that exhibit limited neuronal activity. 2) Furthermore, a sample size of 18 neurons is insufficient to draw strong conclusions. The authors should provide ephys data from a larger number of neurons across multiple culture batches. It would also be informative to examine whether a higher concentration of glutamate (e.g., 100 µM) elicits similar or distinct response profiles in iNeurons. 3) Since the majority of ASD risk genes influence neurodevelopment, using iNeurons, which are relatively immature, as the model may overlook important gene functions related to neuronal maturation.

The reviewer makes valid points – however, the purpose of this manuscript is not to characterize the activity and maturity of iNeurons at baseline (numerous earlier papers have focused on this aspect, and limitations of iNeurons are well established), but instead to provide a proof of principle for a new CRISPR screening modality. We agree that application of this approach to more mature systems will have great potential for biological discovery, and have added this point to our revised discussion - but this is beyond the scope of the present manuscript to implement such experiments.

3. Include the co-culture system in the primary screen. The co-culture condition produced distinct outcomes compared to the monoculture condition, showing low correlation with monocultured neurons (Fig. 4) and different phenotypes of genetic perturbations (Line 340-346). Given these differences, it is important to include co-cultured neurons in the primary screen to capture the full spectrum of gene function. In addition, it would be great to assess the toxicity of UV and glutamate in the co-cultured system.

We agree with the reviewer that differences between co-cultured and monocultured neurons are of biological interest, and we have added this to the discussion. However, as for the previous comment, to conduct those additional experiments is beyond the scope of the present manuscript, since this is a proof of principle for a new technology, not an exhaustive application of the technology to relevant biological systems.

4. CROP-seq data confusion. 1) The authors should identify the cell clusters present in their dataset. It is confusing that they stated neuronal differentiation was not a major contributor to the observed changes, yet cannot distinguish different differentiation stages within their iNeuron cultures. 2) Furthermore, the median cell number per perturbation is only 71.5, which is insufficient for robust analysis and limits the strength of the conclusions. Additional data with increased coverage per perturbation will be necessary.

To address this comment, we conducted extensive additional analyses and added new text and figures to the revised manuscript. 1) We have investigated the cell clusters for maturation markers and also directly investigated expression level changes in bona fide maturation markers for all perturbations. We conclude that none of the perturbation acts simply by accelerating or delaying maturation – rather, some perturbations affect the expression of subsets of maturation-relevant genes, indicating a more specific mechanism of action. 2) While it is true that increasing the number of cells will yield more results (this is true for any single cell profiling experiment), our analysis does detect large numbers of statistically significant DEGs, and the correlation between transcriptomic changes triggered by 2 independent guide RNAs targeting the same gene does suggest robustness of results. Therefore, we believe that the depth of our experiment is adequate within the scope of our manuscript, but we have added the caveat raised by the reviewer to the discussion section of our revised manuscript.

Minor:
1. In Fig. 2b, the dashed line is missing.

We added the missing line.

2. In Line 210, the FDR should be consistent with the figure legend.

We corrected this error.
