# Supplementary material for: A massively parallel CRISPR-based screening platform for modifiers of neuronal depolarization
Source: Nat Commun. 2026 Aug 1;17:9299. doi: 10.1038/s41467-026-75882-0 (PMC13526836; doi:10.1038/s41467-026-75882-0)
Supplement: Supplementary file 4 [file 41467_2026_75882_MOESM3_ESM.pdf]

Description of Additional Supplementary Files

File Name: Supplementary Data 1
Description: CaMPARI2 phenotypes observed in targets with Gene Ontology annotations related to neuronal activity

File Name: Supplementary Data 2
Description: CaMPARI2 phenotypes observed in targets associated with neurological disease

File Name: Supplementary Data 3
Description: sgRNA library design and protospacers

File Name: Supplementary Data 4
Description: Secondary screening sgRNA library

File Name: Supplementary Data 5
Description: Aggregated phenotype scores, FDR, and log fold changes for each target gene in each secondary CaMPARI2 screen

File Name: Supplementary Data 6
Description: Phenotype scores, FDR, and log fold changes for sgRNA in each secondary CaMPARI2 screen

File Name: Supplementary Data 7
Description: List of sgRNA protospacers used in the CROP-seq screen.
